# Electrodeposition of Mn-Co/Polypyrrole Nanocomposites: An Electrochemical and In Situ Soft-X-ray Microspectroscopic Investigation

**DOI:** 10.3390/polym9010017

**Published:** 2017-01-06

**Authors:** Benedetto Bozzini, Patrizia Bocchetta, George Kourousias, Alessandra Gianoncelli

**Affiliations:** 1 Dipartimento di Ingegneria dell’Innovazione, Università del Salento, via Monteroni, 73100 Lecce, Italy; patrizia.bocchetta@unisalento.it; 2 Elettra-Sincrotrone Trieste S.C.p.A. strada statale 14 km 163.5 in Area Science Park, Basovizza, 34012 Trieste, Italy; george.kourousias@elettra.eu (G.K.); alessandra.gianoncelli@elettra.eu (A.G.)

**Keywords:** manganese oxide, polypyrrole, nanocomposites, electrodeposition, coherent diffractive imaging, ptychography

## Abstract

Understanding the lateral variations in the elemental and chemical state of constituents induced by electrochemical reactions at nanoscales is crucial for the advancement of electrochemical materials science. This requires in situ studies to provide observables that contribute to both modeling beyond the phenomenological level and exactly transducing the functionally relevant quantities. A range of X-ray coherent diffraction imaging (CDI) approaches have recently been proposed for imaging beyond the diffraction limit with potentially dramatic improvements in time resolution with chemical sensitivity. In this paper, we report a selection of ptychography results obtained in situ during the electrodeposition of a metal–polymer nanocomposite. Our selection includes dynamic imaging during electrochemically driven growth complemented with absorption and phase spectroscopy with high lateral resolution. We demonstrate the onset of morphological instability feature formation and correlate the chemical state of Mn with the local growth rate controlled by the current density distribution resulting from morphological evolution.

## 1. Introduction

Manganese oxides are popular non-precious oxygen reduction reaction (ORR) catalysts for metal/air batteries [1] owing to their low cost, adequate electrochemical stability and good catalytic activity towards the decomposition of hydrogen peroxide, produced by the ORR 2e¯ pathway, typical for alkaline solutions [2,3,4]. The strong interest in MnO_x_ as an electrode material is also related to its good catalytic activity toward the oxygen evolution reaction (OER) [5,6,7,8] that is required for rechargeable and/or reversible systems. Unfortunately, the ORR electrocatalytic activity of MnO_x_ is generally inferior to that of platinum-based electrocatalysts: it exhibits higher overpotentials and it cannot follow the optimal four-electron pathway [9,10]; moreover, its electrochemical stability is not satisfactory. One of the approaches that have been proposed to improve the ORR electrocatalytic performance of manganese oxides, is based on the addition of metal ions, such as: Ni(II), Ca(II), Mg(II), Bi(III), Cr(III), V(V), Mo(IV), La(III), Co(II) [3,11,12,13,14]. The typical alloying effect is twofold: (i) a desirable increase in the half-wave potential, related to a better activity conferred by the transitional dopants; and (ii) the stabilisation of the intermediate Mn(III)/Mn(IV) phase that enhances the oxygen bond splitting, resulting in a favoured four-electron ORR pathway [3,15,16,17,18]. In particular, Co, Ni and Mg-doping of MnO_x_ catalysts has been also reported to increase the durability of electrodes subjected to accelerated ORR ageing [10,15,19,20,21,22,23]. Among literature, transition metal additions to manganese oxides, binary Mn-Co-O_x_ spinels [10,19,20,23] and MnO_x_/CoO_x_ mixtures [21,22] have been the most extensively studied as ORR catalysts in alkaline solutions, because of their high catalytic activity and good corrosion stability with respect to the pure components. Notwithstanding their electrocatalytic appeal, the practical application of Mn-Co oxides is impaired by their poor electrical conductivity: nevertheless, high-conductivity materials can be fabricated by composite synthesis routes, e.g., by dispersing the metal oxide particles into a polypyrrole (PPy) matrix. The use of PPy for the fabrication of composite ORR electrocatalysts is attractive for the following reasons: (i) it is an excellent support for catalysts both in the form of metal oxides [24] and metallorganic complexes [25,26]; (ii) if appropriately treated, it can act as a source of nitrogen, which is believed to enhance the electrocatalytic activity by forming Mn- or Co-N centers; (iii) it exhibits a good electronic conductivity in the doped form; (iv) it is easy to synthesise by chemical [27] or electrochemical [28] routes. Cong at al. [24,29] were the first to demonstrate the possibility of decreasing the ohmic drop of mixed oxide materials and to protect the catalytic sites against dissolution during ORR operation, by growing composite electrodes by alternating layers of PPy and MnCu or NiCu oxide spinel particles. These workers incorporated the oxide particles—that had been pre-synthesised by thermal decomposition—into electrodeposited PPy. As far as the Mn-Co/PPy system is concerned, a recent paper describes the incorporation of manganese cobaltite nanorods into PPy and investigates the ORR mechanism for these materials [30]. An alternative approach to the incorporation of pre-synthesised particles into electrodeposited PPy is to form both the oxide and the conducting polymer by a fully electrochemical route. This approach has been described for Co/PPy [31,32,33] and Mn/PPy [31,32,34,35,36]. In this paper, we extend this approach to the growth of mixed-oxide/PPy composites, in particular concentrating on an in situ dynamic description of the morphochemical development of the composite material during electrodeposition, based on complementary electrochemical and soft-X ray absorption and fluorescence microspectroscopies.

## 2. Materials and Methods

### 2.1. Electrochemical Measurements

The present section describes the chemicals and equipment employed in the electrochemical fabrication protocols and electroanalytical measurements. The electrodeposition bath used to prepare Mn-Co/PPy nanocomposites contains 0.1 M pyrrole, 25 mM MnCl_2_, 25 mM CoCl_2_, 0.1 M tetra-butyl-ammonium-perchlorate (TBAP) and 1% *v*/*v* H_2_O (ultrapure water with a resistivity of 18.2 MΩ·cm) dissolved in acetonitrile. The small amount of water increases the electropolymerisation rate and improves the mechanical properties, the adhesion and the conductivity of the polymer matrix [33,37]. The electrochemical syntheses were performed using a self-fabricated conventional three-electrode cell with a glassy carbon rod (diameter: 3 mm) working electrode (WE) and a Pt wire spiral (5 cm^2^) as counter electrode (CE) and an aqueous silver/silver chloride (Ag/AgCl (3M): 0.209 V/NHE) as reference electrode (RE), connected to the solution by a salt bridge. The liquid junction potential between aqueous and non-aqueous solution has been found to be negligible. All the potentials are benchmarked against the Ag/AgCl scale.

The electrochemical measurements were performed at room temperature using ParStat (Princeton Applied Research–Ametek, Berwin, PA, USA) and VersaSTAT potentiostats (Princeton Applied Research–Ametek, Berwin, PA, USA). Cyclic voltammetric (CV) analyses have been carried out at glassy carbon electrodes in the range −2 V÷+1.2 V at a scan rate of 100 mV·s^−1^. N_2_ (Rivoira, 5 N) was bubbled for 20 min into the solution before the measurement and an N_2_ blanket was kept above the solution during electrochemistry. The WEs were polished mechanically to a mirror finish before each experiment and subsequently subjected to ultra-sonication in distilled water for 10 min and electrochemical oxidation of impurities in 0.5 M H_2_SO_4_ from 0 to 1.5 V at a scan rate of 100 mV·s^−1^, as recommended in [31]. The counter electrode was cleaned by immersion in concentrated HNO_3_ to remove metal impurities and by annealing in a butane flame to eliminate organic residues.

Mn-Co/PPy nanocomposite electrocatalyst were synthesised by modifying the step-pulsed potential procedure recently adopted for Co/PPy electrodeposition [31,33,37], involving suitable repetitions of the cycle detailed below. The potential values were selected according to the cyclic voltammetric results discussed in Section 3.1.1. This cycle is designed in order to grow a composite consisting of two constituents that are formed anodically (PPy, Mn more oxidised that Mn^2+^) and cathodically (Co°, Mn° and/or precipitated Co^2+^- and Mn^2+^-containing species). The potential program is optimised in view of avoiding large monomer concentration gradients at the electrode–electrolyte interface during electropolymerisation and to minimise the stripping of metal particles during the anodic pulses. The initial step (0 V) does not lead to faradaic reactions, but it is required to relax the compositional double layer. After this relaxation step, a layer of PPy is electrodeposited during the first anodic pulse (1.2 V). During the subsequent cathodic pulse (−1.8 V), reduced Co and Mn or precipitated Co^2+^ and Mn^2+^ species can be incorporated into PPy. In the final anodic step of each cycle (1.2 V), another layer of—possibly Mn-doped—PPy is deposited. Some reference Mn-Co/PPy films have been grown directly onto functionalised TEM (Transmission Electron Microscopy)-grid electrodes, as described in [37].

The electrocatalytic activity of Mn-Co/PPy samples towards ORR was evaluated by linear sweep voltammetric (LSV) measurements in O_2_-saturated (SIAD 6.0) 0.1 M KOH electrolyte under quasi-steady-state conditions (5 mV·s^−1^) at different Rotating Disk Electrode (RDE) rotation speeds. A glassy carbon (GC) electrode (AMEL via S. G.B. De La Salle 4, 20132 Milano, Italy) with Mn-Co/PPy electrodeposit (for details on electrodeposition see Section 3.1) was mounted in a RDE (Parstat Model 2273, Princeton Applied Research–Ametek, Berwin, PA, USA). For reference purposes, the same electrochemical experiments were duplicated with solutions that had been de-oxygenated by N_2_ saturation. O_2_ was bubbled for 20 min into the solution before the measurements and an O_2_ blanket was maintained above the electrolyte during voltammetry. The ORR Levich slopes have been evaluated by using current-voltage curves from which the N_2_-background had been subtracted. The current densities are referred to the geometric area. 

### 2.2. Soft-X-ray Fluorescence and Absorption Microspectroscopies

Soft X-ray fluorescence (XRF) elemental mapping was performed at the TwinMic beamline of Elettra synchrotron facility (Trieste, Italy) [38,39]. The photon beam was focused to a microprobe using zone plate optics and the imaging measurements were performed by simultaneous detection of transmitted and emitted (fluorescence) photons raster-scanning the sample with respect to the microprobe. The transmitted photons were detected using a fast readout CCD (Charge Coupled Device) camera (Andor Technology Ltd., Belfast, Northern Ireland), generating absorption and phase contrast images [40,41], whereas the emitted fluorescence signal was collected by means of 8 Silicon Drift Detectors (SDDs) located in an annular geometry in front of the specimen [42,43]. XAS (X-ray Absorption Spectroscopy) spectroscopy can also be applied on specific points of interest by using a photodiode located downstream the specimen. For the specific experiment shown in this paper XRF mapping was combined with absorption imaging. Photon energies in the ranges: 635–648 eV and 764–790 eV were chosen to set the best excitation conditions for Mn and Co and well as to measure stacks of absorption images, as detailed in Section 3.3.3. The XRF elemental mapping was performed at 920 eV in order to excite both Co and Mn: The responses of each of the 8 SDDs was recorded and summed together. The deconvolution of the fluorescence spectrum for each pixel was performed with the PyMCA software package [44], by using the Hypermet algorithm and linear baseline subtraction. Final data processing was carried out by Matlab (Version 6.5.0, The MathWorks Inc., Natick, MA, USA). The L emission lines of Mn (637.4 eV) and Co (797.0 eV), and the elastic scattering peak were monitored along the scanned area. The post size of the beam was chosen between 1.5 μm and 250 nm according to the size of the feature of interests. It is worth emphasizing that, under the operating conditions adopted for this study, we did not assess any measurable radiation damage effect.

## 3. Results and Discussion

### 3.1. Electrodeposition of Mn-Co/PPy Composites

#### 3.1.1. Cyclic Voltammetric Study

**Figure 1:**
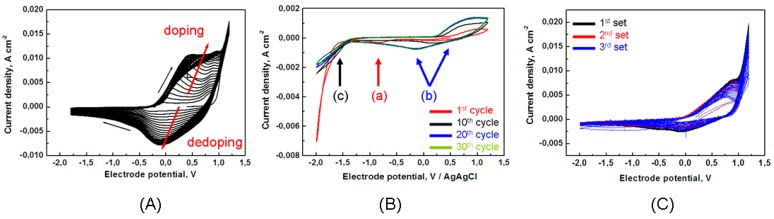
Cyclic voltammograms corresponding to: (**A**) pyrrole polymerisation; (**B**) codeposition of Mn and Co; (**C**) codeposition of polypyrrole, Mn and Co (three independent replicated experiments). Glassy carbon (GC) electrode in contact with acetonitrile/water 1 vol % solutions containing 0.1 M tetra-butyl-ammonium-perchlorate (TBAP) with added: (**A**) 0.1 M pyrrole; (**B**) 0.025 M MnCl_2_ and 0.025 M CoCl_2_; (**C**) 0.1 M pyrrole, 0.025 M MnCl_2_ and 0.025 M CoCl_2_. Scan rate 100 mV·s^−1^.

Figure 1 reports cyclic voltammograms (CV) corresponding to (A) pyrrole polymerization, (B) Mn-Co electrodeposition and (C) Mn-Co/PPy co-electrodeposition from the solutions detailed in Section 2.1.

##### 3.1.1.1. Electrodeposition of Pyrrole

In Figure 1A, we plot the CVs for the electropolymerisation of pyrrole in 0.1 M TBAP and 1% *v*/*v* H_2_O de-aerated acetonitrile (ACN) solution. The first cycle follows the typical nucleation loop of conducting polymers [45] and the electrode process begins on the polymer-free GC surface at potentials higher than 0.9 V, coherent with previous works [31,32]. In the successive cycles, two waves appear: The first one during the positive-going scan at ~0–0.4 V and the second one during the cathodic scan at ~0÷−0.2 V, corresponding, respectively, to the doping/un-doping processes of the PPy film with perchlorate anions [32,46,47].

##### 3.1.1.2. Electrodeposition of Mn-Co

In order to better understand the incorporation process of Mn and Co species into PPy during composite electrosynthesis, the electrochemical behaviour of mixed MnCl_2_/CoCl_2_ containing solution in absence of pyrrole has been investigated. The voltammetric analysis of the electrolytic solutions containing separately CoCl_2_ or MnCl_2_ can be found in previous works of ours: [32,34,36], respectively. Briefly, on the one hand, CV experiments conducted in CoCl_2_-containing solution [32] have shown the typical cathodic nucleation loop and anodic stripping peak of the metals. On the other hand, the electrochemical behavior of MnCl_2_-containing bath [34] was found to be more complex, as a result of different valence states for manganese that can prevail in the relevant electrochemical conditions used for our experiments. The CVs indicate that Mn(II) species oxidise mainly to Mn(III, IV) oxide/hydroxide in the anodic scan and reduce to Mn(0) in the cathodic one. The electroreduction of Mn metal goes on through an irreversible process with a large difference (ca. 1 V) between the Mn(II)/Mn(0) reduction peak and the corresponding anodic one, as observed in literature for 0.066 M MnCl_2_/dimethyl formamide solution on Pt [48]. In our solution containing water, electrodeposited Mn metal is not thermodynamically stable and undergoes chemical oxidation by H_2_O producing Mn^2+^ (low pH) or Mn(OH)_2_/HMnO_2_^−^ (high pH) [49]. This is confirmed by soft X-ray absorption spectroscopy of Mn/PPy samples, that have shown that Mn is present only in oxidised valence states: (II, III, IV) [34].

Figure 1B reports the CVs recorded in the range +1.2–−2 V with solutions containing both MnCl_2_ and CoCl_2_ as the electroactive species. In the first cycle, one can notice a cathodic peak (1th cycle, peak (a)), that can be attributed to the reduction of the Co(II) complex present in the solution to Co(0), and the absence of a correspondent stripping wave: It is worth noting that such a stripping peak is recorded at −0.05 V when only CoCl_2_ is present in the solution ([32] and references therein contained). 

The irreversibility of the Co reduction peak is probably related to the fact that Co becomes covered by Mn(0) and/or by Mn/Co oxyhydroxide electroreduced or electroprecipitation in the high-cathodic range of the CVs [50,51]. In fact, the reduction of water (2 H_2_O + 2e^−^ → H_2_ + 2 OH¯)—Producing a local increase of pH—Is thermodynamically possible as the equilibrium standard potential in acetonitrile is reported to be 0 V/NHE [52]. Both Mn(II) and H_2_O reduction occurs at about −1.6 V in the first cycle and at more positive potentials (−1.3 V) in the subsequent ones (peak (c)). The gradual disappearance of the peak (a) (related to the formation of Co(II)/Co(0)) with cycling, is coherent with the behaviour of the same bath containing only CoCl_2_, where the decrease of both Co(0) deposition and stripping waves were observed upon successive CV scans. Since this phenomenon has not been observed in aqueous solution containing the same electrolytes CoCl_2_/TBAP [32], we can argue that the decisive role in the behaviour of Co plating/stripping CV waves is played by the coordination structures of Co complexes with acetonitrile.

In the anodic-going scan of the first potential cycle one can notice that a broad anodic wave appears at ca. 0.6 V, followed by an apparent diffusion-controlled plateau. The onset potential of this wave is the same as that observed in baths containing only MnCl_2_, while the shape is significantly different. This behaviour can be ascribed to the possible anodic formation of mixed Mn-CoO_x_ or Co-doped MnO_X_ [9,53], the CV behaviour of which can replace or partially overlap with (or kinetically modify) the typical one of Mn(III, IV) oxides features, ascribed to the multi-step process illustrated in [35,36,54].

The subsequent scans are very similar to those reported for electrolytes containing only MnCl_2_, with the exception that: (i) broader features are found when both metals salts and (ii) a new cathodic peak appears at −0.55 V (peak (b)). As in the case of the pure Mn electrolyte, the anodic broad wave at ca. 0.6 V tends to grow in the anodic-going scans and in correspondence, a new cathodic peak located at 0.3 V appears after the 1st cycle. This cathodic peak associated to MnOOH/Mn(II) reduction in MnCl_2_ electrolyte, gradually shifts to more negative potentials (ca. −0.55 V) upon cycling, suggesting that the reduction process in MnCl_2_/CoCl_2_ bath involves the formation of mixed Mn/Co oxide.

##### 3.1.1.3. Electrodeposition of PPy/Mn-Co

In Figure 1C, we report three independent replicated CV curves obtained with the bath containing pyrrole, MnCl_2_ and CoCl_2_. The anodic electron-transfer processes corresponding to the formation of Mn(III, IV) oxides and Mn-Co mixed oxides and the doping of PPy with perchlorate anions (Figure 1A) run in the same potential range. Moreover, it can be observed that the reduction processes of Mn(II) to Mn(0) and OH¯ generation occurring at ca. −1.5 V in the absence of pyrrole (Figure 1B), are suppressed by the presence of the polymer, owing to the increase in ohmic drop caused by the un-doping process. At variance with the curves obtained in the solution free from Mn^2+^ and Co^2+^ (Figure 1A), the un-doping process (ClO_4_¯ expulsion) of PPy is less evident with respect to the doping one, indicating that the incorporation of Mn species in the polymer during the anodic scan modifies its electronic structure. The modifications of the doping and un-doping curve revealed in Panel C can be interpreted by considering that MnCl_2_ and CoCl_2_ are able to form anionic complexes in ACN solvent and can thus be incorporated during the anodic electropolymerisation of pyrrole together with perchlorate anions. The incorporation of catalytic sites as counteranions by the polymerisation of pyrrole in a medium containing the anionic catalyst as the supporting electrolyte is a possible route to fabricate PPy-based catalytic electrodes [55,56,57]. Specifically, [58] demonstrated the incorporation of cobalt, iron and manganese porphyrin, iron and cobalt phthalocyanines, as well as cobalt(II) salts in PPy by doping with the relevant anions dissolved in the solution. In addition, Pt nanoparticles have been embedded into PPy films by doping with PtCl_4_^2−^ anions during the electropolymerisation and, subsequently, reduction of the anions within the polymer film [59]. Of course, CV analyses alone are not able to definitively establish the valence state of Mn and Co inside the polymer due to the complex electrochemistry of the bath and the presence of a multiplicity of oxidation and reduction processes (especially for Mn). For this reason, a multitechnique has been devised in this work, in order to elucidate the chemical distribution of the species inside the PPy matrix.

#### 3.1.2. Analysis of the Potentiostatic Chronoamperograms Recorded during Composite Synthesis

##### 3.1.2.1. Anodic Potensiostatic Pulse

**Figure 2:**
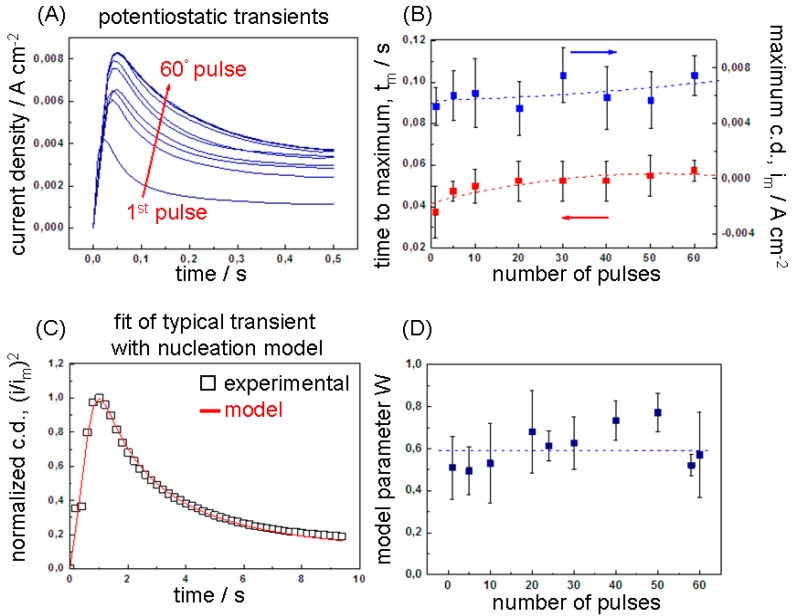
(**A**) Potentiostatic transients for the anodic growth of Mn-Co/PPy on a graphite electrode in contact with acetonitrile/water 1 vol % solutions containing 0.1 M TBAP, 0.1 M pyrrole, 0.025 M MnCl_2_ and 0.025 M CoCl_2_ at the applied potential of 1.2 V(Ag/AgCl); (**B**) Maximum current density (*i*_m_) and the corresponding time (*t*_m_) as a function of the number of the sequential pulses recorded during the electrosynthesis; (**C**) Dimensionless plot of a typical transient (the 45th one), compared with theoretical mixed nucleation curve [Mele 09]; (**D**) *W* values obtained by best fitting of each anodic transient. The *i*_m_, *t*_m_ and *W* values reported on the plot are averaged (the error bar is the standard deviation) on four independent experimental data sets.

Figure 2A shows the experimental current transients observed during the pulsed step potentiostatic synthesis of the Mn-Co/PPY samples. The shape of the curves with a well-defined current maximum (*t*_m_, *i*_m_) is typical of the nucleation and growth of a new solid phase on an electrode [60,61,62,63,64,65,66] and can be studied with the nucleation and growth theory developed by Scharifker-Hills [67,68,69,70,71,72]. As discussed in Section 3.1.1, the possible anodic processes occurring at the applied electrode potential of 1.2 V are: (i) electropolymerisation of pyrrole; (ii) oxidation of the Mn(II) complexes in the bath and/or of the Mn(II, III) and Co(0) species already incorporated in PPy and (iii) polymer doping with ClO_4_¯ and possibly with anionic complexes of Mn(II) and Co(II). These electrode reactions lead to the electrodeposition of a solid composite material made of PPy and Mn/Co oxyhydroxides. All of these three kinds of electrode reactions are able to induce a nucleation and growth process at the electrochemical interface for: (i) PPy electropolymerisation [18,73,74,75] as well as (ii) anodic electrodeposition of MnO_x_ [76] and (iii) CoO_x_ [61]. Moreover, similar transient shapes have been demonstrated for PPy doping [77,78,79].

In Figure 2B, we report the maximum current density (*i*_m_) and the corresponding time (*t*_m_) as a function of the number of the sequential pulses recorded during the electrosynthesis. The *t*_m_ values ranging from 37 to 50 ms are in agreement with those reported for the electropolymerisation of pyrrole on GC [18] (0.06–0.15 s), highly oriented pyrolytic graphite (HOPG) [74,75] (0.1–0.2 s) and vitreous carbon [73] (0.026–0.15 s). It can be noticed that both parameters slightly increase with the pulse number (with more rapidity from the 1st to the 20th pulse). The increase of *t*_m_, associated to a decrease of the number of generated nuclei, can be explained by considering that the effective applied anodic potential slightly decreases with the progress of the pulses. It is worth noting that the rate at which the PPy oxidises (incorporates anions) at every anodic pulse is influenced by the preceding cathodic pulse, i.e., by the degree of compactness of the polymeric structure attained during the expulsion of anions. As studied in detail in [77,78,79], the electrochemical doping/un-doping processes involve not only the interface electrode–electrolyte, but the entire polymer volume, resulting in conformational and relaxation variations during the electrochemical switching between the reduced (insulating/compact) and oxidised (electronically conducting/relaxed) PPy states. Accordingly, the relaxation time required for the oxidation (doping) of the polymer increases when increasing the time (*t_w_*) of cathodic pre-polarization (un-doping) [80]. By considering a reasonable doping efficiency during the anodic pulses, the portion of un-doped polymer (and thus *t_w_*) gradually increases at every pulse together with the time required to re-dope the polymer. This results in a slight decrease in polymer electronic conductivity with the pulse cycles and, thus, in the effective applied potential (*E*_eff_ = 1.2 V −η_ohm,PPY_(*n*_pulse_)). A possible contribution to this effect could arise from the incorporation of non-conductive Mn/Co oxy-hydroxides. As demonstrated by previous studies based on micro-X-ray absorption spectroscopy, metallic Co can be also incorporated, but in lower quantity [32], while metallic Mn is not present [34,36]. The increase of *i*_m_ values can be related to three factors: (i) the gain of the effective surface area of the electrode due to the appearance of a number of fresh nuclei at every pulse; (ii) a possible increase of the growth rate of the nuclei pre-formed in the previous anodic pulse at higher potential [81]; (iii) the increase of the electropolymerisation kinetics related to the progressive PPy coverage of the electrode surface [32]. The traditional use of *i-t* curves to extract mechanistic information, is to discriminate between instantaneous and progressive nucleation and assigning numerical values for *N_s_* and *AN_o_*, respectively. These two types of nucleation are in fact limiting cases of a more general situation in which, at any given time, nuclei both form and grow. A simple way of accounting for this is to formulate models able to accommodate with continuity a family of curves intermediate between the two limiting cases. This can be achieved e.g., by decoupling the parameters *A* and *N_o_* and defining an analytical form of the transient in which *A* can vary over the whole positive real semiaxis (e.g., [69,82,83]). The conceptual clarity of this approach, as well as the possibility of estimating *A* and *N_o_* from a single transient, are in practice counteracted by added numerical complexities that in fact end up recoupling the two parameters. A simpler approach was proposed in [84], based on the linear combination of the two limiting cases of instantaneous and progressive nucleation with a weight *W*: 0 ≤ *W* ≤ 1 [84]. *W* gives a measure of the distance from the limiting cases (*W* = 0 progressive nucleation, *W* = 1 instantaneous nucleation) and provides a single-parameter, physically straightforward description of the shape changes of *i-t* transients. The only other free parameter *t*_m_ can be interpreted simply by referring to both (weighted) limiting nucleation cases. Moreover, it can be straightforwardly proved that the weighted equation can be regarded as an approximation of the *i-t* curves proposed in the references quoted above in this section. Accordingly, the current density and time of the experimental current transients have been normalised according to the coordinates of current maximum: (*i*_m_, *t*_m_) and compared to the theoretical *i*/*i*_m_ vs. *t*/*t*_m_ curves [84] in Figure 2C. The values of *W* have been determined through an iterative fitting process according to the protocol reported in [85] and plotted as a function of the number of pulses in Figure 2D. The results show that the instantaneous and progressive mechanisms equally contribute to the nucleation process during the application of the pulse train.

##### 3.1.2.2. Cathodic Potensiostatic Pulse

**Figure 3:**
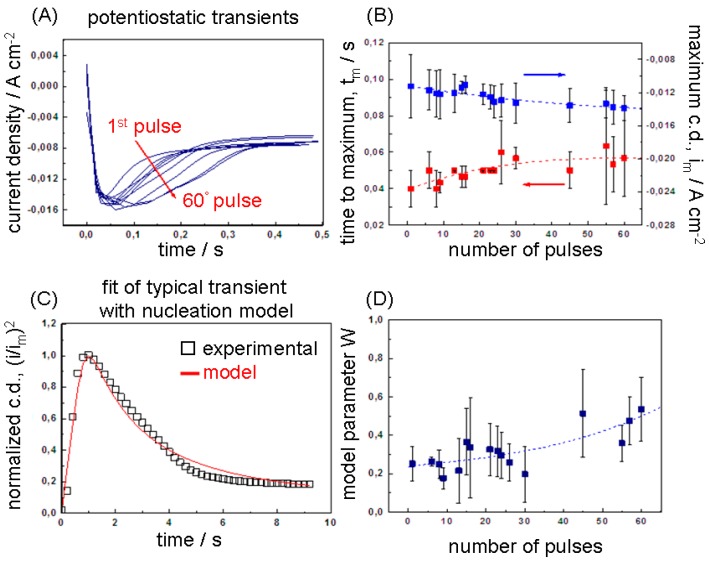
(**A**) Potentiostatic transients measured during the cathodic growth of Mn-Co/PPy on a graphite electrode in contact with acetonitrile/water 1 vol % solutions containing 0.1 M TBAP, 0.1 M pyrrole, 0.025 M MnCl_2_ and 0.025 M CoCl_2_ at the applied potential of −1.8 V(Ag/AgCl); (**B**) Maximum current density (*i*_m_) and the corresponding time (*t*_m_) as a function of the number of the sequential pulses recorded during the electrosynthesis; (**C**) Dimensionless plot of a typical chronoamperogram (the 45th one), compared with the theoretical mixed nucleation curve [Mele 09]; (**D**) *W* values obtained by best fitting of each cathodic chronoamperogram. The *i*_m_, *t*_m_ and *W* values reported on the Panels (B,D) are averaged (the error bar is the standard deviation) on four independent experimental data sets.

Figure 3A shows the experimental cathodic chronoamperograms observed during the pulsed step potentiostatic synthesis of the Mn-Co/PPy samples at the applied cathodic potential of −1.8 V. As for the anodic pulse, the shape of the transients shows a rapid increase in current up to a maximum, followed by a current drop characteristic of cathodic nucleation and growth of a solid material on a conducting surface. The electrodeposited nuclei are made of a composite material, the nature of which is determined by the possible cathodic processes: (i) reduction of the Mn(II) and Co(II) complexes in the bath to Mn(0) and Co(0); (ii) cathodic reduction of the Mn(III)/Mn(IV) and Co(II) oxides species already incorporated into PPy; (iii) polymer un-doping and (iv) cathodic electroprecipitation of Mn and Co oxy-hydroxides. The nucleation of a solid phase on the electrode surface, well-known for the electrodeposition of metals (reaction (i)), has been also observed during the precipitation of oxy-hydroxides under electrogeneration of base (reaction (iv)) [86]. The maximum current density (*i*_m_) and the corresponding time (*t*_m_) are plotted as a function of the pulse number as shown in Figure 3B. The slight increase of *t*_m_, as discussed for the anodic chronoamperograms, can be related to the gradual increase in the ohmic drop of polymer, resulting in a corresponding lowering of the effective applied cathodic potential. The progressive increase of maximum cathodic current density (*i*_m_) with the pulse number can be due to the increase of the effective surface area of the electrode due to the cumulative growth of new nuclei and to the roughening of the PPy surface, influenced by the conformational compactness of the polymer during the un-doping process, i.e., perchlorate anion expulsion [79]. The *W* values have been determined as detailed for in Section 3.1.2.1 and plotted as a function of the number of pulses (Figure 3D). Inspection of *W* values shows that the nucleation mechanism is mainly progressive and the instantaneous contribution increases with the pulse number until the end of the pulse train where the two contribute equally (*W* = ~0.5).

Of course, the simultaneous presence of instantaneous and progressive nucleation processes and their relative weight during pulse plating could be fully understood by only taking into account the complex interplay of electrochemical, chemical and polymerisation processes. In fact, the nucleation mechanism is strongly dependent on the nature of the growing material as well as on the features of the substrate and on the properties of the electrolyte. As discussed above, different electrochemical processes operate simultaneously during the same anodic and cathodic nucleation processes. Furthermore, the chemical nature of Mn-Co/PPy nuclei and the prevailing electrodeposition kinetics change with the pulse number due to the variability of the electrocatalytic properties of the electrode surface depending on the Mn-Co species already deposited, with possible activation of concurrent reactions such as H_2_ evolution as well as on the electrical and conformational properties of the PPy, resulting in changes of porosity and electrical field distribution.

The dynamic interplay of complex chemical conditions expounded above calls for a multitechnique approach, sensitive to the space distribution of the valence of the elements involved in view of a comprehensive understanding of the relevant electrodeposition process.

### 3.2. Oxygen Reduction Reaction (ORR) Electrocatalytic Performance

**Figure 4:**
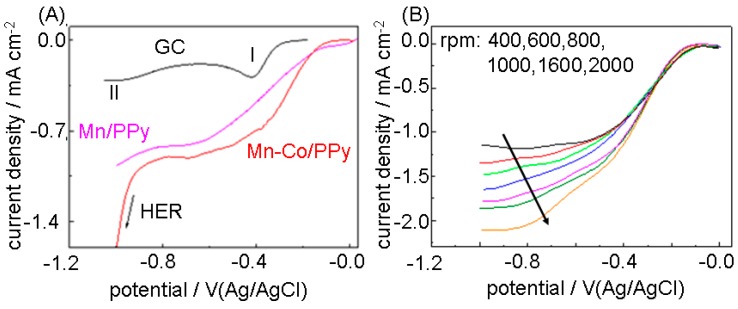

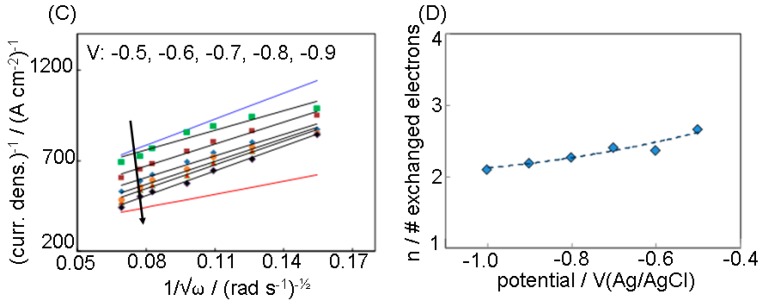
(**A**) LSVs curves recorded at Mn/PPy/GC, Mn-Co/PPy/GC and GC electrode in 0.1 M O_2_-saturated 0.1 KOH solution at 5 mV·s^−1^; (**B**) Rotating Disk Electrode (RDE) voltammograms (scan rate: 5 mV·s^−1^) of Mn-Co/PPy electrodeposited on GC in O_2_-saturated 0.1 M KOH at different rotation rates. The N_2_ background has been subtracted; (**C**) Koutecky–Levich plots derived from the curves of Panel (**C**) at a series of representative potentials; (**D**) Dependence of the electron transfer number *n* on the electrode potential for Mn-Co/PPy samples.

The electrocatalytic activity towards ORR of as-electrodeposited Mn-Co/PPy samples was investigated following the protocol described in Section 2.1 Figure 4A reports the ORR LSV curves of Mn-Co/PPy, compared to those corresponding to the Mn/PPy composite electrodeposited under otherwise identical conditions and to the bare GC support. Of course, the presence of M/PPy (M = Mn and/or Co) on GC drastically improves the ORR electrocatalysis with respect to bare GC in terms of: (i) onset potential (*E_onset_*); (ii) half wave potential (*E_1/2_*) and (iii) nominal current density. The ORR curves recorded on GC typically exhibit two waves (I and II in Panel A) both attributed to the 2-electron transfer reduction of O_2_ producing H_2_O_2_ [87]. The values of onset potentials are quite similar for Mn-Co/PPy (*E_onset_* = −0.115 V), Mn/PPy (*E_onset_* = −0.085 V) and Co/PPy (*E_onset_* = −0.1 V [32]) and close to the value of −0.09 V recorded for polycrystalline bulk Pt in the same conditions [32]. The beneficial effect of Co addition to Mn/PPy on the ORR can be observed from the half wave potential increase from *E_1/2_* = −0.364 V (Mn/PPy) to −0.280 V (Mn-Co/PPy), the latter appearing negatively shifted by 110 mV with respect to the bulk Pt data measured in a previous work of ours (*E_1/2_* = −0.17 V) [32].

In Figure 4B, we report ORR quasi-steady-state voltammograms of as-electrodeposited Mn-Co/PPy samples at a scan rate of 5 mV·s^−1^ in O_2_-saturated 0.1 KOH aqueous solutions at different RDE rotation speeds. The presence of a non-perfectly flat plateau in the high-current density region of these cyclovoltammetric curves is often observed in M/N/C electrocatalytic materials [31,88,89,90,91] and has been treated theoretically. This phenomenon can be associated with the heterogeneity of the surface, impeding the attainment of pure diffusion-limited conditions due to the presence of a distribution of catalytic activity on the surface. The formation of a multiplicity of catalytically active sites can be followed by in situ microspectroscopy measurements, as detailed in Section 3.3.

The electron transfer number *n* was determined by the Koutecky-Levich equations [92]:

(1) $$\frac{1}{j}=\frac{1}{j_{L}}+\frac{1}{j_{K}}=\frac{1}{B\sqrt{\varpi }}+\frac{1}{j_{K}}$$

(2) $$B=0.62nFC_{o}{\left(D_{o}\right)}^{\frac{2}{3}}\nu^{-\frac{1}{6}}$$

where: *j_K_* is the kinetic current density; *j_L_* the diffusion–limiting current density; *j* the measured current density; *B* the reciprocal of the slope; ϖ the angular velocity of the disk; *F* the Faraday constant, *C*_0_ the saturation concentration of O_2_ in 0.1 M KOH at room temperature (1.2 × 10^−6^ mol·cm^−3^); *D*_0_ the diffusion coefficient of oxygen in water (1.73 × 10^−5^ cm^2^·s^−1^) and *v* the kinematic viscosity of the solution at room temperature (0.01 cm^2^·s^−1^) [93]. The Koutecky-Levich plots are reported in Figure 4C. The slopes of their linear fit lines were used to estimate *n* according to Equation (2) in a range of electrode potentials that are representative of practical electrocatalytic operation. The *n* values, averaged in the potential range—0.5–−1 V, were found to be 2.35 ± 0.197, suggesting the prevalence of ORR two-electron mechanism on the four-electron reduction to water. Similar values are reported for Mn_3_O_4_/C (*n* = 2.4), MnO/C (*n* = 2.2) and MnO_2_/C (*n* = 2.3) in the same potential range [94]. As shown in Figure 4D, higher *n* values are found in the low overpotential range: a similar behaviour has been reported for MnO_x_/C electrocatalysts [94], where the Koutecky–Levich electron number *n* approaches 2 at high overpotentials and higher rotation rates and 4 at low overpotentials (at ca. 0.106 V) and low rotation rates. Since as-electrodeposited PPy is ORR inactive [31], the knowledge of the chemical-state of Mn and Co in the composite and its correlation with the electrodeposition parameters is key to optimising the synthesis process toward more efficient electrocatalysts.

### 3.3. In Situ X-ray Fluorescence (XRF) Mapping and Scanning X-ray Absorption Microscopy (SXM)

Elemental and chemical-state distributions during electrodeposition were followed in situ by XRF and SXM, respectively. Chemical-state sensitivity was achieved by acquiring stacks of absorption images by scanning the beam energy across the Co and Mn L_3_-edges. XRF and SXM images were collected after having applied appropriate numbers of electrodeposition cycles, highlighting the morphochemical changes taking place between the initial stages of nanocomposite growth and the final condition of an electrocatalyst layer of practical interest (typically between 50 and 120 cycles).

#### 3.3.1. Cell Fabrication

**Figure 5:**
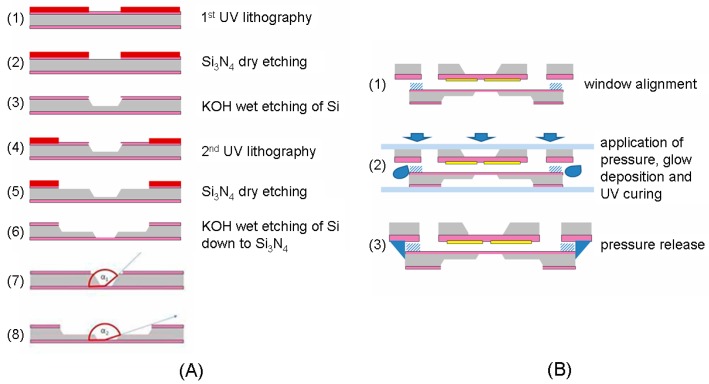
(**A**) After the definition of suitable pattern by proximity UV lithography (1), the silicon nitride film has been etched by the reactive ion etching process. A first KOH step (2) etches a groove in the Si wafer of dimension close to that of the final Si_3_N_4_ window. A second lithographic step (3), followed by dry-etching (4), defines the frame pattern. The final KOH etch (5) produces the Si_3_N_4_ window supported on a Si frame of controlled thickness (500 nm). Panels (7) and (8) show the difference in signal emission angle of between the conventional narrow window (7) and the novel wide one (8); (**B**) Cell-assembly scheme. The procedure consists in: (1) alignment of the Si_3_N_4_ windows; (2) application of a controlled pressure, addition of NOA 84 and cross-linking by UV exposure, after which the final cell configuration (3) is obtained.

The fabrication process of the liquid cells starts from the production of 100 nm thick Silicon Nitride (Si_3_N_4_) windows by standard KOH etching (33% in weight, 90 °C) (for more details, see [33]). Every cell consists of two silicon chips exhibiting different window patterns. In the first chip, we fabricate three windows, two of which are employed as an inlet and outlet for electrolyte injection. The third one, located in an intermediate position, is manufactured with a novel process (Figure 5A) allowing to obtain a soft X-ray transparent window consisting of a 1 × 1 mm^2^ Si_3_N_4_ membrane surrounded by a 2 × 2 mm^2^ Si frame with a thickness that is locally smaller than that of the wafer (100 against 500 μm). This window geometry allows the collection of signal also at grazing angle (see Panels (7) and (8) of Figure 5A). The electrodes were obtained by a standard lift-off process consisting in a first Proximity ultraviolet (UV) Lithography step (PMGI SF3\S1813 resist bilayer was used), thermal evaporation of a Cr/Au metal film (thicknesses 5 and 40 nm, respectively) and subsequent stripping of resists in hot acetone and MF319 develop. Of course, any material suitable to be deposited with a lithographic approach can be used in our cell concept and the choice of electrodes is no way limited to Au. The sealed reservoir for electrolyte containment with controlled height was obtained by joining the two silicon chips through a multistep fabrication procedure, consisting in a first step of deposition and patterning of convenient negative resist (SU8-3000.5) by UV lithography on one part to produce the channel of liquid path. The second chip has been put in contact and joined under low pressure with a UV curable resin (NOA 84) in order to ensure the sealing action (Figure 5B). The spacing between the two optical windows, defining the thickness of the electrolyte layer, is 500 nm. After injection of the electrolyte, the inlet and outlet ports are closed by gluing thin glass slide pieces by Araldite adhesive, providing perfect sealing and negligible solvent emission under high vacuum condition.

#### 3.3.2. Dynamic XRF Mapping

**Figure 6:**
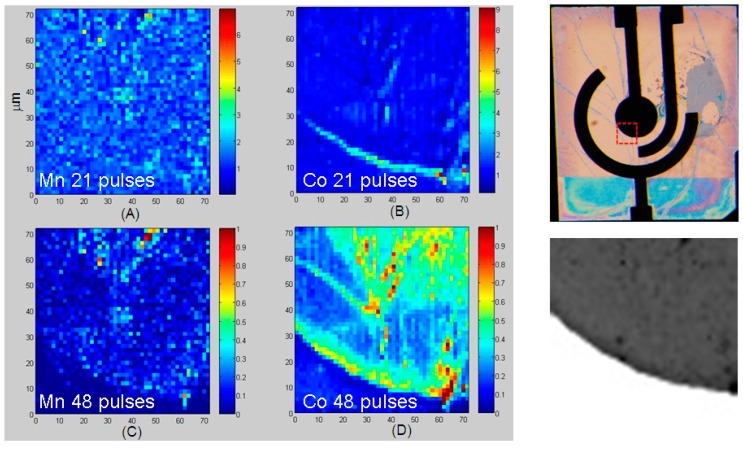
Soft X-ray fluorescence (XRF) Mn (**A**,**C**) and Co (**B**,**D**) high-lateral resolution maps (1.5 μm spot size), elaborated as indicated below. (**A**,**B**) Maps measured after 48 growth steps divided by the corresponding maps recorded after 21 growth steps; (**C**,**D**) Maps measured after 48 growth steps (normalised). All XRF maps are normalised with respect to the corresponding scattering map. On the right-hand side of the figure, an optical micrograph of the whole electrodic system is shown, with the indication of the analysed zone, alongside with a representative absorption image of the working electrode (acquired at 920 eV after 10 growth steps).

In order to assess the progressive build-up of Mn and Co during the growth of Mn-Co/PPy composites, XRF mapping was performed after the application of appropriate sequences of electrodeposition steps. Owing to experimental time constraints, only two conditions were compared at high lateral resolution, while several growth steps were monitored at lower resolution. In Figure 6, we report Mn (Panels A and C) and Co (Panels B and D) high-lateral resolution XRF maps measured in situ after 21 (Panels A and B) and 48 cycles (Panels C and D), together with an optical micrograph of the whole electrodic system of the micro-cell showing the location of the analysed zone, alongside with a representative absorption image of the same zone. The accumulation of the two elements with electrodeposition time and their time-dependent distribution can be appreciated. As demonstrated in [33], the current density is higher at the electrode–electrolyte border and it progressively decreases going inside the electrode. A difference in space distribution is found between Co and Mn that can be explained on the basis of the electrokinetic peculiarities of the two metals. Since the build-up of Co is almost entirely controlled by electroreduction of Co(II), a higher concentration of Co is found close to the WE border (Panels B and D), following the secondary current density distribution at the electrode–electrolyte interface. Mn instead is chiefly incorporated via basic salt precipitation triggered by electrochemical alkalinisation and the latter element is relatively more homogeneously distributed (panels A and C). In fact, alkalinisation is controlled by a reaction-diffusion process for which the source term is localized at the electrode–electrolyte interface, but precipitation also occurs in the bulk, following the hydroxide concentration gradient.

**Figure 7:**
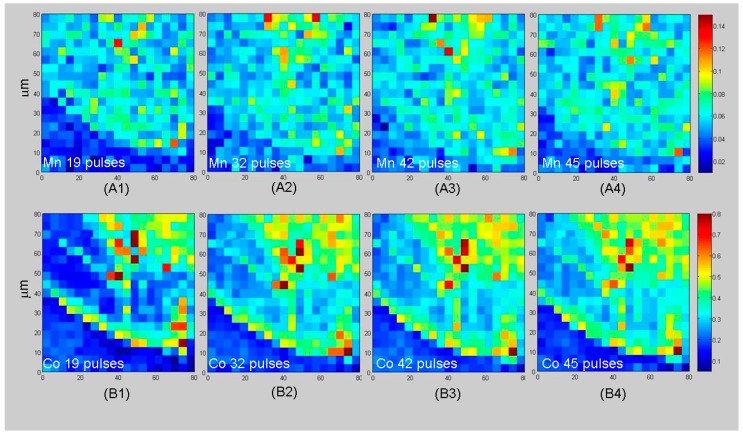
XRF Mn (**A1**–**A4**) and Co (**B1**–**B4**) low-lateral resolution maps (4.0 μm spot size), elaborated as indicated below. Maps measured after: (1) 19, (2) 32, (3) 42 and (4) 45 growth steps, normalised with respect to the corresponding scattering maps.

The patterns forming in the internal region of the WE are controlled by reaction-diffusion processes and can be followed with the DIB electrodeposition model (see [95,96,97] and references therein contained). Non-linear least squares fitting of experimental data with the DIB model has been recently demonstrated and will be the subject of a dedicated publication. The progress of the electrodeposition process can be even more clearly appreciated from Figure 7, which depicts a sequence of XRF Mn and Co maps recorded after 19, 32, 42 and 45 electrodeposition steps. The images reported in Figure 7 are at lower space resolution than those shown in Figure 6 and contain the raw data without any smoothing or interpolation: this is physically important, even though it is at the cost of cosmetic appeal, because artefacts can be introduced but such image processing actions alter the physical message. Such lower space resolution is dictated by synchrotron beamtime restrictions—Beyond aesthetics—That does not impair the physical message of the measurement. It is worth noting that also this pattern development can be followed with the DIB model, which is able to predict a morphochemical transient evolving towards steady-state patterns, coherent with the time-behaviour displayed by the succession of panels.

#### 3.3.3. Chemical-State Mapping with Stacks of SXM Absorption Images

**Figure 8:**
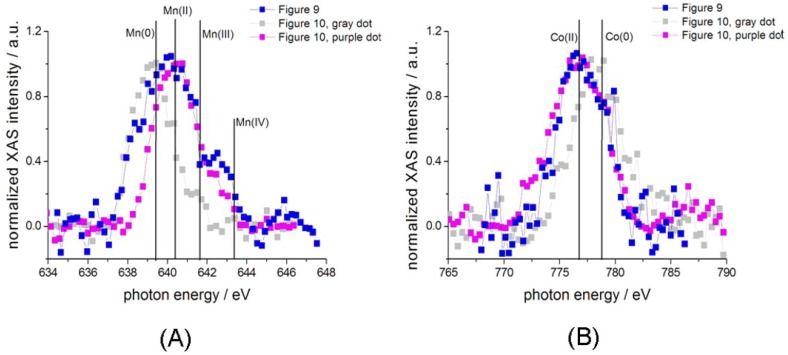
(**A**) Mn L_2_ and (**B**) Co L_3_ XAS spectra extracted from stacks of images and corresponding to one pixel. The positions of the relevant pixels are shown in Figure 9 and Figure 10.

**Figure 9:**
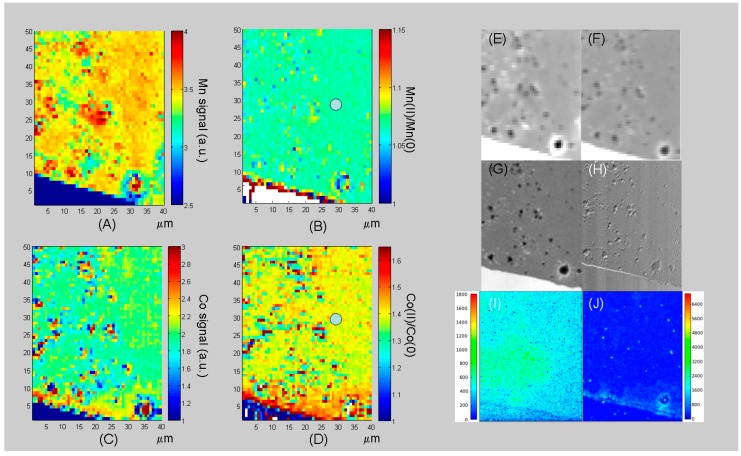
(**A**–**D**) Images reconstructed from stacks of SXM images recorded in situ across the Mn (**A**,**B**) and Co (**C**,**D**) L_3_-edges after 120 electrodeposition cycles. Elemental distributions (1 μm spot size diameter) of Mn (**A**) and Co (**C**). Mn(II)/Mn(0) (**B**) and Co(II)/Co(0) (**D**) ratios; (**E**,**F**) Examples of SXM images (1 μm spot size diameter) extracted from the stack, at the Mn and Co L_3_-edge, respectively; (**G**,**H**) High-lateral resolution (200 nm spot size diameter) SXM images ((**G**) absorption contrast, (**H**) phase contrast: the vertical stripes are due to synchrotron beam instabilities) of the same region, recorded at 920 eV and (**I**,**J**) the corresponding high-lateral resolution (200 nm spot size diameter) Mn and Co XRF maps, respectively, acquired at 920 eV. The grey dot indicates the position to which the XAS spectra reported in Figure 8 correspond.

**Figure 10:**
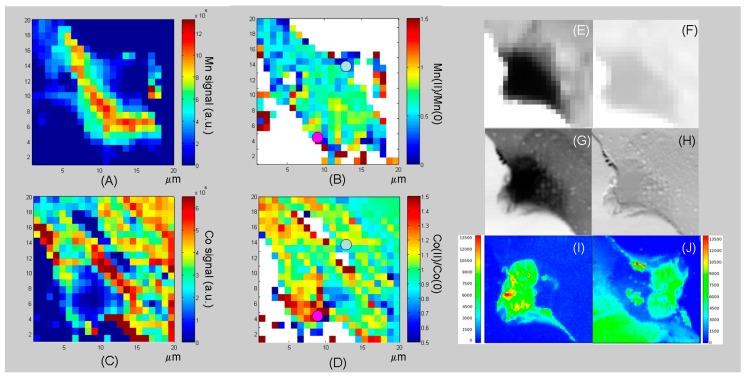
(**A**–**D**) Images of a dendrite reconstructed from stacks of SXM images recorded in situ across the Mn (**A**,**B**) and Co (**C**,**D**) L_3_-edges after 120 electrodeposition cycles. Elemental distributions (1 μm spot size diameter) of Mn (**A**) and Co (**C**). Mn(II)/Mn(0) (**B**) and Co(II)/Co(0) (**D**) ratios; (**E**,**F**) Examples of SXM images (1 μm spot size diameter) extracted from the stack, at the Mn and Co L-edge, respectively; (**G**,**H**) High-lateral resolution (250 nm spot size diameter) SXM images ((**G**) absorption contrast, (**H**) phase contrast) of the same region, recorded at 920 eV and (**I**,**J**) the corresponding high-lateral resolution (250 nm spot size diameter) Mn and Co XRF maps, respectively, acquired at 920 eV. The grey and purple dots indicate the positions to which the XAS spectra reported in Figure 8 correspond. The grey spot is at the root of the dendrite, while the purple one is at its tip.

By using the SXM imaging mode, it is possible to extract elemental and chemical-state information at high lateral resolution. According to this approach, conventional SXM images are acquired while scanning the energy across relevant absorption edges: the resulting stack of images encodes absorption contrast changes with the beam energy, which allows to extract an absorption spectrum for each pixel of the micrograph. In this case, we analysed in situ the WE after the growth of a catalyst layer resulting from the application of 120 electrodeposition cycles by scanning the energy across the Mn L_2_ and Co L_3_-edges in steps of 0.25 eV. In Figure 8, we report a selection of representative Mn and Co spectra extracted from these stacks of images, further analysed in Figure 9 and Figure 10. We investigated the electrode edge (Figure 9) as well as a dendrite that has developed as a result of the rather prolonged growth time (Figure 10). Panels A–D of both figures report a selection of results of the elaboration of the stacks of absorption images, while the other panels provide complementary information. In particular: (i) Panels E and F show a typical SXM image from the stack, corresponding to the L_3_ absorption edge; (ii) Panels G and H report, respectively, high-lateral resolution absorption and phase-contrast images of the same region and (iii) Panels I and J the Mn and Co XRF maps acquired during the measurements of image (G). Of course, the stacks of images contain a large amount of information and can be elaborated in several ways in order to extract compositional and chemical-state maps. As far as the spectral features of the Co L_3_ edge XAS are concerned (Figure 8A), it should be noted that whereas Co(0) is characterized by a single principal feature at ca. 778.7 eV and Co(II) shows two main peaks at 776.7 and 778.0 eV, Co(III) exhibits two principal bands at 780.2 and 782.3 eV. The Mn L_2_ XAS spectra (Figure 8B) have distinct features for different Mn oxidation states, some of them being at very close photon energies (Mn(0) ca. 639.3 eV, Mn(II) ca. 640.5 eV, Mn(III) ca. 641.7 eV, Mn(IV) ca. 643.5 eV), so they can overlap in the case of coexistence of several oxidation states. In particular, none of the spectra exhibits the pure line shape of a single Mn oxidation state, but typically consist of two or three overlapping components. Owing to the fact that the spectral features of the Mn L_2_ edge systematically shift to higher photon energies with an increased oxidation state, the principal components can be extracted for mapping purposes in a reliable way. In the present study, we concentrated on: (i) elemental distribution (Mn and Co: Panels A and C of Figure 9 and Figure 10) and (ii)—Since the peaks essentially exhibit two components corresponding to the elemental M(0) and divalent M(II) forms of the metal, coherent with [33,35]—The ratio M(II)/M(0), Mn(II)/Mn(0) and Co(II)/Co(0): Panels B and D of Figure 9 and Figure 10). Still more specifically, restricting chemical state, mapping to the M(II)/M(0) ratio does not lead to a loss of information since the trend of the oxidation states with current density is a systematic one, that confirms the findings of [33,35]. The elemental distributions were obtained by peak integration and the chemical-state distribution by rationing the intensities of the appropriate peak components over suitable regions of interest. By comparing Panels A and C of Figure 9 and Figure 10, one can notice that more Mn than Co is found in higher current density regions, coherently with the electrodeposition mechanism of each metal, recalled above in Section 3.3.2. Similarly, analysis of Panels B and D shows higher Mn(II)/Mn(0) and Co(II)/Co(0) ratios in higher current density regions, matching with the findings of [33,36], respectively; it is worth emphasising that the results of these papers were based on the collection of a series of local absorption spectra in selected positions of the WE rather than on each pixel of the image as in the present investigation. Regions characterised by differences in local current density exhibit different proportions of elemental and oxidized Co. In particular, from Figure 9 and Figure 10, it is evident that higher current density areas are richer in oxidised Co while lower current density ones are enriched in the elemental form. This is a result of the combined anodic/cathodic electrodeposition process necessary for the formation of the relevant composite. This can be simply explained by the fact that, from the employed solution, Co can only be brought into the solid phase as Co(0) during the cathodic steps of the potentiostatic sequence, while polypyrrole forms during the anodic steps; during the anodic steps, Co, originally deposited as Co(0), partially oxidises. During subsequent cathodic steps, in principle, depending on the activity of these species, oxidised Co present in the film can transform back to the metallic state. Thus, in the high-current density areas where the oxidation rate is higher, the formation of Co(II) is favoured. Furthermore, inspection of the chemlocal state maps shows that, in the high current density areas, Co tends to form well-defined particles, coherent with high growth rate conditions. In these regions, Co can be measured only in the particles, while no Co signal is measured in other locations corresponding to the flat zones of the electrode. In intermediate current density areas, Co appears more uniformly distributed over the electrode. The XAS intensities correlate well with the absorption contrast: the highest intensities found in the intermediate current density region are about the same as those of the hot-spots of the high current density zone. Notably less intense Co spectra, chiefly corresponding to Co(0), are obtained in the low current density areas. Upon inspection of the distributions of Mn and Mn(II)/Mn(0) ratios, one can clearly see that differences in current density induce inhomogeneities both in the amount and in the chemical state of electrodeposit. Moving from lower to higher current densities, there is a systematic shift to higher photon energies, indicative of the fact that higher current densities favour the deposition of oxidised forms of Mn, with a predominance of Mn(II) with respect to Mn(0). Notwithstanding the fact that Mn(II) can be formed both by electrochemical oxidation of pre-deposited Mn(0) and by precipitation of Mn(II) basic salts (see Section 3.3.2 above) meaning that the distribution of Mn(II) is more homogeneous, which is the explanation given for Co of the correlation between M(II)/M(0) and current density that also applies to Mn. The steeper current density gradients present on the dendrite illustrated in Figure 10 explain the higher heterogeneity in elemental and chemical-state distributions found in this unstable outgrowth feature with respect to the smoother region analysed in Figure 9.

## 4. Conclusions

The present research expounds the electrochemical fabrication of Mn-Co-O_x_/polypyrrole nanocomposites for electrocatalytic applications relevant to metal-air battery technology, and contributes to the mechanistic understanding of the pulsed electrodeposition process. Our study is based on a multi-technique approach combining electrochemical measurements and complementary in situ soft-X-ray fluorescence and absorption microspectroscopies, allowing an understanding of time- and space-dependent phenomena leading to nanocomposite formation.

As evidenced by cyclovoltammetric measurements, the anodic electron-transfer processes corresponding to the formation of Mn(III, IV) oxides and Mn-Co mixed oxides as well as the doping of PPy with perchlorate anions occur under the same electrochemical conditions, giving rise to synergistic effects that are not found in the electrochemical synthesis of the individual components of the composite, as well as to a modification in the polymer electronic structure, related to co-doping with perchlorate, Mn(II) and Co(II). In particular, high-cathodic processes (formation of Mn(II) and Mn(0) as well as base generation) are inhibited by polymer un-doping. Anodic and cathodic potentiostatic transients—Corresponding to the oxidation and reduction branches of the composite synthesis protocol, respectively—Show a progressive decrease in the number of generated nuclei, accompanied by an enhancement of the transient current maximum. The former process is a result of the fact that the rate at which the PPy incorporates anions at every anodic pulse is influenced by the preceding cathodic pulse, that in turn controls the structural compaction driven by anion expulsion; while the latter phenomenon results, on the one hand from surface area growth resulting from progressive nucleation, and on the other hand from the anodic growth-rate enhancement of polymer nuclei generated in foregoing oxidation cycles. The overall transient current behaviour correlates with a slight, but measurable decrease in polymer electronic conductivity with the pulse number. Finally, in the cathodic and anodic pulses, the prevailing nucleation mechanisms were found to be progressive and instantaneous, respectively. As far as the ORR catalytic performance is concerned, the beneficial effect of Co can be observed from a notable half wave potential increase with respect to Mn/PPy. Moreover, the electron transfer number *n* was to be ca. 2.3, corresponding to a prevailing two-electron O_2_ reduction mechanism.

The build-up of the elemental distribution of Mn and Co during the growth of Mn-Co/PPy composites could be followed by in situ XRF mapping. The space and time distribution of the two electrodeposited elements is coherent with the electrodeposition mechanisms of Mn and Co in this system (precipitation of the formed, direct reduction of the latter), controlled on the one hand by the current density distribution ensured with electrode design and on the other hand by reaction-diffusion processes that yield spatio-temporal pattern formation. The analysis of stacks of absorption images acquired in situ by scanning the beam energy across the Mn and Co L_3_-edges was used to image the elemental (Mn and Co) and chemical-state (M(II)/M(0)) distributions prevailing on the one hand in a relatively homogeneous region of the working electrode and on the other end on a dendrite. We found that more Mn than Co is found in higher current density regions, in line with the findings of our XRF maps. Similarly, higher Mn(II)/Mn(0) and Co(II)/Co(0) ratios were measured in higher current density regions, confirming—Though with a much higher lateral resolution—The observations and mechanistic interpretations proposed in previous works of ours centred on the coelectrodeposition of the individual metals with PPy, based on in situ local XAS.
